# Association of endothelial activation assessed through endothelin-I precursor peptide measurement with mortality in COVID-19 patients: an observational analysis

**DOI:** 10.1186/s12931-021-01742-8

**Published:** 2021-05-13

**Authors:** Claudia Gregoriano, Dominik Damm, Alexander Kutz, Daniel Koch, Selina Wolfisberg, Sebastian Haubitz, Anna Conen, Luca Bernasconi, Angelika Hammerer-Lercher, Christoph A. Fux, Beat Mueller, Philipp Schuetz

**Affiliations:** 1 Medical University Department of Medicine, Kantonsspital Aarau, Tellstrasse, CH-5001 Aarau, Switzerland; 2 University of Basel, Basel, Switzerland; 3 Department of Infectious Diseases and Hospital Hygiene, Kantonsspital Aarau, Switzerland; 4 Institute of Laboratory Medicine, Kantonsspital Aarau, Aarau, Switzerland

**Keywords:** COVID-19, Endothelin-1, All-cause 30-day mortality, ProET-1, Prognostic marker, Risk assessment, SARS-CoV-2

## Abstract

**Background:**

Severe acute respiratory syndrome coronavirus 2 (SARS-CoV-2) disease (COVID-19) has been linked to thrombotic complications and endothelial dysfunction. We assessed the prognostic implications of endothelial activation through measurement of endothelin-I precursor peptide (proET-1), the stable precursor protein of Endothelin-1, in a well-defined cohort of patients hospitalized with COVID-19.

**Methods:**

We measured proET-1 in 74 consecutively admitted adult patients with confirmed COVID-19 and compared its prognostic accuracy to that of patients with community-acquired pneumonia (n = 876) and viral bronchitis (n = 371) from a previous study by means of logistic regression analysis. The primary endpoint was all-cause 30-day mortality.

**Results:**

Overall, median admission proET-1 levels were lower in COVID-19 patients compared to those with pneumonia and exacerbated bronchitis, respectively (57.0 pmol/l vs. 113.0 pmol/l vs. 96.0 pmol/l, p < 0.01). Although COVID-19 non-survivors had 1.5-fold higher admission proET-1 levels compared to survivors (81.8 pmol/l [IQR: 76 to 118] vs. 53.6 [IQR: 37 to 69]), no significant association of proET-1 levels and mortality was found in a regression model adjusted for age, gender, creatinine level, diastolic blood pressure as well as cancer and coronary artery disease (adjusted OR 0.1, 95% CI 0.0009 to 14.7). In patients with pneumonia (adjusted OR 25.4, 95% CI 5.1 to 127.4) and exacerbated bronchitis (adjusted OR 120.1, 95% CI 1.9 to 7499) we found significant associations of proET-1 and mortality.

**Conclusions:**

Compared to other types of pulmonary infection, COVID-19 shows only a mild activation of the endothelium as assessed through measurement of proET-1. Therefore, the high mortality associated with COVID-19 may not be attributed to endothelial dysfunction by the surrogate marker proET-1.

## Background

Coronavirus disease 2019 (COVID-19), a novel human pathogenic severe acute respiratory syndrome coronavirus counts meanwhile worldwide more than 60 million confirmed cases and more than 1.4 million deaths [1]. Severe acute respiratory syndrome coronavirus 2 (SARS-CoV-2) was first recognized in December 2019 in Wuhan (China) [2]. Infected patients present with a broad spectrum of symptoms causing a mild to critical disease [3]. Most of the patients (81%) show mild infections while severe disease was reported in 14% and critical disease in 5%, depending on the specific patient population [3]. Most frequent symptoms associated with COVID-19 are cough, fever, myalgia, headache, dyspnea, sore throat, gastrointestinal symptoms as well as loss of smell or taste [4]. Particularly, respiratory symptoms are common, because SARS-CoV-2 enters host cells via protein angiotensin-converting enzyme 2 (ACE2), which is expressed by alveolar epithelial type II cells and makes the lungs a main target for infection [5, 6]. Since many other extra-pulmonary tissues, including heart, kidney, liver and intestine, express ACE2 receptors as well, [7] symptoms associated with these organs, especially acute myocardial injury, kidney failure and diarrhea, can occur [8, 9].

Another target of SARS-CoV-2 infections is the endothelium, which also expresses ACE2 [10, 11], leading to endothelial dysfunction as a major determinant of COVID-19. This may lead to a loss of physiological properties of the endothelium, including the ability to stimulate vasodilation, fibrinolysis, and anti-aggregation [12]. Previous research found that endothelial dysfunction plays an important role in critical illness, especially in sepsis [13].

Endothelin-1 (ET-1), mainly synthesized by activated endothelial cells which are stimulated by bacterial endotoxin and various inflammatory cytokines (TNF-alpha, interleukin-6), is a potent vasoconstrictor agent [14]. Further, ET-1 is one of the major endogenous factors controlling vasotonus [15], which is responsible for blood pressure homeostasis and blood supply to individual organs [13, 16–18]. Because of the instability of ET-1 at room temperature and its rapid clearance from circulation, the more stable precursor fragment of ET-1, called C-terminal proendothelin-1 (proET-1), which can be measured by a sandwich immunoassay [19], is the preferable biomarker to assess endothelial dysfunction. At present, there is a lack of clinical data regarding the release of ET-1 in COVID-19 patients. As SARS-CoV-2 is targeting the endothelium, we hypothesize that circulating ET-1 levels are increased during acute illness and correlate with the risk for 30-day all-cause mortality in patients with COVID-19 requiring hospitalization. This study aimed to compare levels of proET-1 in patients with COVID-19 with those of patients with other types of respiratory infections and to investigate its association with all-cause 30-day mortality.

## Methods

### Study design and setting

This prospective observational study included all consecutively hospitalized adult patients (≥ 18 years) with confirmed COVID-19 at the Cantonal Hospital Aarau, a tertiary care medical center in Switzerland, between February 26, 2020 and April 30, 2020.

Baseline characteristics of the analyzed COVID-19 patients have been published elsewhere [20]. In brief, COVID-19 was defined by a positive real-time reverse transcription polymerase chain reaction (RT-PCR) taken from nasopharyngeal swabs or lower respiratory tract specimens according to the WHO guidelines [21] and typical clinical symptoms of the presenting patients (e.g., respiratory symptoms with or without fever, and/or pulmonary infiltrates and/or anosmia/dysgeusia). The study was approved by the ethical committee (EKZN, 2020-01306) and performed in conformance with the Declaration of Helsinki ethical guidelines. All analyzed data were assessed as part of the clinical routine during the hospitalization.

### Data collection

Clinical data, including socio-demographics, comorbidities as well as pre-existing medical prescription and COVID-19-specific inpatient medication were assessed until hospital discharge or death and extracted from the electronic health records. Experimental antiviral treatment was recorded if given and included hydroxychloroquine (alone or in combination with azithromycin) and sometimes tocilizumab. Comorbidities were also assessed via chart review and based on the International Statistical Classification of Diseases and Related Health Problems codes (ICD10). Additionally, patient outcomes including admission to the intensive care unit (ICU), length of hospital stay (LOS) as well as length of ICU stay were collected via chart review. 30-day mortality was collected by abstraction of hospital records, or where necessary by systematic telephone interviews. Laboratory test results were available according to clinical routine. ProET-1 was batch-tested at the end of the study and was therefore not available to the treating team during the index hospitalization.

### Control group

We used patients with confirmed community-acquired pneumonia or acute and chronic exacerbated bronchitis included in a previous prospective study as a control group [22, 23]. The results of this study analyzing proET-1 have been reported in detail elsewhere [24]. In brief, from October 2006 to March 2008 consecutive patients with respiratory infection from six different hospitals located in the northern part of Switzerland were included and prospectively followed-up for the assessment of mortality and other endpoints.

### Endpoint and study objective

The primary endpoint of this investigation was all-cause 30-day mortality. For the COVID-19 patients and the control group, we assessed vital status 30 days after admission by abstraction of hospital records and/or systematic telephone interviews.

### Measurements of proET-1

Plasma and serum samples on admission and during follow-up were collected in BD Vacutainer® Heparin and SST tubes. Routine left-over samples were stored at −80 °C until analysis. Results from routine laboratory tests were recorded. C-terminal proendothelin-1 (CT-proET-1) was assessed in batch using a commercially available automated fluorescent sandwich immunoassay (KRYPTOR™, B.R.A.H.M.S Thermo Fisher Scientific, Germany), as described in detail elsewhere [25, 26]. The immunoassays have a limit of detection (LOD) of 2.94 pmol/l [27]. The functional assay sensitivity, defined as the concentration with an inter-assay coefficient of variation of < 20%, is 9.78 pmol/l. Values for the analytes followed a Gaussian distribution in healthy individuals without significant differences between males and females [26]. The laboratory technicians who performed the measurements were blinded to the characteristics of the patients and the characteristics of the study.

For the COVID-19 affected patients various time points during hospitalization were analyzed, depending on the available data:

- T_0_ (initial blood draw upon hospital admission)
- T_1_ (day 3/ day 4)
- T_2_ (day 5/ day 6)
- T_3_ (day 7/ day 8)

For the control group, blood samples for later marker measurement were collected upon admission, i.e., within the first 24 h upon hospital admission. ProET-1 levels were batch-measured in plasma with sandwich immunoassays (Kryptor® Thermo Scientific Biomarkers) [23].

### Statistical analysis

Discrete variables are expressed as frequency (percentage) and continuous variables as medians with interquartile ranges (IQR) or mean with standard deviation (SD). In addition to descriptive statistics, we investigated the association of proET-1 levels at different time points with the primary endpoint by means of multivariable logistic regression analysis with reporting of odds ratios (OR) and corresponding 95% confidence intervals (CI) and p-values as a measure of association. As predefined, three types of regression models were calculated, namely an unadjusted model, a model adjusted for age and gender and a model adjusted for age, gender, creatinine level, diastolic blood pressure and comorbidities. The confounders were based on previous reports published by Bhandaria et al. [28] as well as based on medical knowledge. The number of confounders was limited to avoid over-fitting of the regression models. Laboratory values with non-normal distribution were log-transformed before entering the statistical models. C-statistics was calculated as a measure of discrimination. We also validated the prognostic value of different predefined proET-1 cut-offs to predict all-cause 30-day mortality according to different proET-1 cut-offs as already presented in other non-COVID-19 studies. More specifically, one study showed the best cut-off point regarding sensitivity and specificity in predicting ICU mortality at 74 pmol/L [14]. Another study showed an optimal proET-1 cut-off of 94 pmol/L for mortality, and 154 pmol/L for prediction of bacteremia [29]. Additionally, we assessed sensitivity, specificity, positive and negative predictive values of different proET-1 cut-offs to predict 30-day mortality. Groups were compared with Wilcoxon rank sum test. A two-sided *p*-value of < 0.05 was considered significant. Statistical analysis was performed using Stata 15.1 (StataCorp, College Station, TX, USA).

## Results

**Fig. 1:**
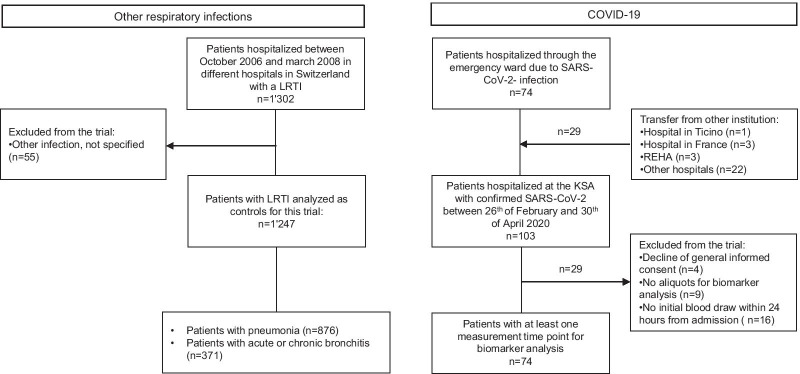
Flow chart of the study. Abbreviations: KSA, cantonal hospital Aarau; LRTI, lower respiratory tract infection; SARS-CoV-2, severe acute respiratory syndrome coronavirus 2

Overall, we included 103 patients with confirmed COVID-19 (74 were admitted directly and 29 were transferred from other hospitals). Four patients were excluded from the analysis (decline of general consent). Further 25 cases had to be excluded because of missing aliquots for biomarker analysis (n = 9), or due to missing blood sampling within 24 h from admission (n = 16). Thus, the final analysis encompassed 74 COVID-19 patients. In addition, 1247 control patients from a previously conducted study were used with a diagnosis of pneumonia (n = 876) or acute or chronic exacerbated bronchitis (n = 371). Figure 1 provides an overview of the study flow.

### Baseline characteristics

Table 1 shows patient demographics, comorbidities as well as vital signs, laboratory findings and outcomes within 30 days in all analyzed patients and stratified by type of respiratory infection. Overall, COVID-19 patients were younger and more often male compared to the control groups. Further, patients with COVID-19 had higher initial blood pressure values with median systolic blood pressure of 141.5 mmHg (IQR 126.0 to 156.0 mmHg), and diastolic blood pressure of 81.5 mmHg (IQR 72.0 to 88.0 mmHg) as compared to both control groups (p < 0.01). In general, COVID-19 patients had a higher prevalence of comorbidities. Initial laboratory findings were similar in patients with COVID-19 and controls, except for results of blood gas analysis. Patients affected by COVID-19 showed significantly lower values of PCO_2_ with (32 mmHg (IQR 31 to 35 mmHg) and without initial O_2_-supply (31 mmHg (IQR 29 to 33 mmHg) at admission compared to the control patients (p < 0.01). Regarding 30-day outcomes, COVID-19 patients had higher rates of all-cause mortality and admission to ICU. Length of stay was also longer in COVID-19 patients.

**Table 1:** Baseline characteristics and 30-day endpoints for the analyzed study population stratified by respiratory infections

	Pneumonia (n = 876)	Acute or chronic exacerbated bronchitis (n = 371)	SARS-CoV-2 (n = 74)	*p*-value
Sociodemographics				
Age [years] median (IQR)	73.0 (59.0, 82.0)	73.0 (60.0, 81.0)	64.5 (57.0, 74.0)	< 0.01
Male gender, n (%)	361 (41.2%)	168 (45.3%)	47 (63.5%)	< 0.01
Pre-existing risk-factors and medication				
Smoker	219 (25.7%)	123 (34.1%)	4 (7.0%)	< 0.01
Steroid intake	69 (8.1%)	69 (18.9%)	1 (1.4%)	< 0.01
Immunosuppressive therapy	14 (1.6%)	3 (0.8%)	3 (4.1%)	0.11
Comorbidities				
Cancer, n (%)	110 (12.6%)	42 (11.3%)	6 (8.1%)	0.48
Coronary artery disease, n (%)	176 (20.1%)	85 (22.9%)	18 (24.3%)	0.42
Chronic heart failure, n (%)	151 (17.2%)	55 (14.8%)	2 (2.7%)	< 0.01
Solid organ transplant recipient, n (%)	2 (0.2%)	1 (0.3%)	0 (0.0%)	0.91
Chronic kidney disease, n (%)	196 (22.4%)	79 (21.3%)	18 (24.3%)	0.82
Liver cirrhosis, n (%)	19 (2.2%)	4 (1.1%)	0 (0.0%)	0.20
Obesity (BMI > 30 kg/m2), n (%)	35 (17.7%)	32 (18.4%)	23 (31.1%)	0.04
Initial vital signs				
Blood pressure, systolic [mmHg], median (IQR)	132.0 (119.0, 148.0)	138.0 (120.0, 150.0)	141.5 (126.0, 156.0)	< 0.01
Blood pressure, diastolic [mmHg], median (IQR)	74.0 (65.0, 82.0)	78.0 (66.2, 85.0)	81.5 (72.0, 88.0)	< 0.01
Heart rate [bpm], median (IQR)	95.0 (82.0, 107.0)	90.0 (80.0, 101.0)	87.5 (77.5, 97.5)	< 0.01
Respiratory rate [breaths/min], median (IQR)	20.0 (16.0, 25.0)	20.0 (16.0, 26.0)	22.0 (18.0, 27.0)	0.39
Temperature [°C], median (IQR)	38.1 (37.2, 38.9)	37.3 (36.7, 38.1)	37.7 (37.2, 38.3)	< 0.01
Temperature > 38 °C, n (%)	446 (50.9%)	105 (28.3%)	37 (50.0%)	< 0.01
SpO_2_ [%], median (IQR)	93.0 (89.0, 96.0)	93.0 (89.0, 96.0)	93.0 (87.8, 95.3)	0.99
Initial laboratory findings				
Blood gas analysis for ambient air, n (%)	n = 455	n = 198	n = 54	
FiO_2_ [%], median (IQR)	21	21	21	N/A
PO_2_ [mmHg], median (IQR)	62 (57, 76)	66 (58, 76)	68 (61, 72)	0.9
PCO_2_ [mmHg], median (IQR)	35 (31, 38)	36 (32, 41)	31 (29, 33)	< 0.01
Lactate [mmol/l], median (IQR)	1.6 (1.0, 2.3)	1.4 (1.1, 1.6)	1.3 (0.9, 1.7)	0.1
Blood gas analysis for initial O_2_, n (%)	n = 421	n = 173	n = 20	
FiO2 [%], median (IQR)	32 (28, 36)	28 (28, 36)	47 (32, 95)	< 0.01
PO_2_ [mmHg], median (IQR)	60 (53,70)	62 (53, 74)	65 (56, 75)	0.24
PCO_2_ [mmHg], median (IQR)	35 (31, 39)	38 (34, 47)	32 (31, 35)	< 0.01
Lactate [mmol/l], median (IQR)	1.3 (1.0, 1.8)	1.0 (0.9, 1.4)	1.2 (1.0, 1.5)	0.11
Leukocytes [G/L], median (IQR)	12.0 (9.0, 16.4)	9.7 (7.5, 13.3)	7.3 (4.6, 8.7)	< 0.01
Sodium [mmol/L], median (IQR)	136.0 (133.0, 138.0)	136.0 (134.0, 139.0)	136.0 (133.0, 139.0)	< 0.01
Glucose [mmol/L], median (IQR)	7.1 (6.0, 8.5)	6.7 (5.8, 7.9)	6.7 (5.9, 8.6)	0.01
Urea [mmol/L], median (IQR)	7.1 (4.9, 10.5)	6.5 (4.4, 9.6)	5.9 (4.4, 9.4)	0.06
CRP [mg/L], median (IQR)	154.5 (74.2, 251.9)	41.0 (14.0, 98.4)	94.3 (49.9, 150.0)	< 0.01
PCT [µg/L], median (IQR)	0.46 (0.15, 2.66)	0.12 (0.08, 0.20)	0.11 (0.05, 0.26)	< 0.01
Creatinine [µmol/L], median (IQR)	89.0 (69.0, 113.0)	84.0 (66.0, 106.0)	91.0 (77.0, 115.0)	0.02
Pro-Endothelin-1 [pmol/L], median (IQR)	113 (81.0, 169.0)	96.0 (67.0, 139.0)	57.0 (41.4, 81.8)	< 0.01
Outcomes within 30 days				
ICU care, n (%)	80 (9.1%)	13 (3.5%)	22 (29.7%)	< 0.01
30-day mortality, n (%)	49 (5.6%)	10 (2.7%)	17 (23.0%)	< 0.01
Length of stay [day], median (IQR)	8.0 (5.0, 13.0)	7.0 (3.0, 11.0)	9.0 (5.0, 14.0)	< 0.01

BMI, Body-Mass-Index; bpm, beats per minute; C, Celsius; CRP, C -reactive protein; FiO_2_, fraction of inspired oxygen; ICU, intensive care unit; IQR, interquartile range; mmHg, millimeter of mercury; NA, not applicable; pCO_2_, partial pressure of carbon dioxide; PCT, procalcitonin; PO_2_, partial pressure of oxygen; SpO_2_, oxygen saturatio

### Association of ProET-1 and 30-day mortality

Median proET-1 was lower in COVID-19 patients compared to pneumonia and exacerbated bronchitis patients, respectively (57.0 pmol/l vs. 113.0 pmol/l vs. 96.0 pmol/l, p < 0.01). Still, among all patient groups, non-survivors had higher proET-1 levels at admission compared to survivors (Table 2). In the COVID-19 population, non-survivors had 1.5-fold higher median admission proET-1 levels compared to survivors (81.8 pmol/L (IQR 76 to 118 pmol/L) vs. 53.6 pmol/L (IQR 37 to 69 pmol/L)). In patients with acute or chronic exacerbated bronchitis initial proET-1 levels were 1.8-fold higher in non-survivors when compared to patients who survived (176.5 pmol/L vs. 94 pmol/L) and in patients with pneumonia proET-1 levels were 1.9-fold increased (209 pmol/L vs. 110 pmol/L). Table 3 gives an overview of results from regression analyses and C-statistics. While proET-1 was associated with mortality in the COVID-19 population in the unadjusted analysis, this was no longer significant in the multivariable analysis adjusted for age, gender, creatinine level, diastolic blood pressure as well as cancer and coronary artery disease. A subgroup analysis regarding age, gender and comorbidities also showed no evidence for effect modification. However, for patients with pneumonia and acute or chronic exacerbated bronchitis, proET-1 was more strongly associated with mortality and associations also remained statistically significant in multivariable analyses. Regarding unadjusted discrimination for 30-day mortality, proET-1 had highest accuracy for acute or chronic exacerbated bronchitis (AUC 0.83), but was similar for pneumonia (AUC 0.75) and for COVID-19 patients (AUC of 0.73).

**Table 2:** ProET-1 values and different cut-offs stratified by the analyzed respiratory infections and by survivors and non-survivors

	Pneumonia (n = 876)		Acute or chronic exacerbated bronchitis (n = 371)		SARS-CoV-2 (n = 74)	
	Survivors (n = 827)	Non-Survivors n = 49)	Survivors (n = 361)	Non-Survivors (n = 10)	Survivors (n = 57)	Non-Survivors (n = 17)
proET-1 values						
proET-1 overall [pmol/L], median (IQR)	110.0 (80.0, 163.0)	209.0 (135.0, 328.0)	94.0 (67.0, 133.0)	176.5 (155.0, 230.0)	53.6 (37.0, 69.0)	81.8 (76.0, 118.0)
proET-1 cut-offs, n (%)						
< 74 [pmol/L]	162 (19.6%)	3 (6%)	114 (31.6%)	0 (0%)	44 (77.2%)	4 (24%)
≥ 74 [pmol/L]	141 (17.0%)	4 (8%)	68 (18.8%)	1 (10%)	8 (14.0%)	8 (47%)
> 94 [pmol/L]	292 (35.3%)	9 (18%)	115 (31.9%)	1 (10%)	3 (5.3%)	3 (18%)
> 154 [pmol/L]	232 (28.1%)	33 (67%)	64 (17.7%)	8 (80%)	2 (3.5%)	2 (12%)
proET-1 median-cut-off, n, (%)						
< 107 [pmol/L]	378 (45.7%)	9 (18%)	215 (59.6%)	1 (10%)	52 (91.2%)	12 (71%)
≥ 107 [pmol/L]	449 (54.3%)	40 (82%)	146 (40.4%)	9 (90%)	5 (8.8%)	5 (29%)

IQR, interquartile range; proET-1, proEndothelin-1; SARS-CoV-2, severe acute respiratory syndrome coronavirus 2

**Table 3:** Logistic regression analyses forAssociation of initial proET-1 values and 30-day mortality in the different respiratory infections

	Pneumonia (n = 876)	Acute or chronic exacerbated bronchitis (n = 371)	SARS-CoV-2 (n = 74)
Regression analysis, OR^c^ (95% CI), p-value			
Unadjusted model	51.1 (15.0 to 174.2), p < 0.01	215.3 (10.9 to 4246.2), p < 0.01	32.2 (2.3 to 455.5), p = 0.01
Multivariable model 1^a^	36.7 (9.7 to 138.4), p < 0.01	82.0 (2.8 to 2394.0), p = 0.01	6.6 (0.2 to 193.3), p = 0.27
Multivariable model 2^b^	25.4 (5.1 to 127.4), p < 0.01	120.7 (1.9 to 7499.7), p = 0.02	0.1 (0.0009 to 14.7), p = 0.38
Discrimination statistics AUC (95% CI)			
AUC (95% CI) Unadjusted model	0.75 (0.67 to 0.83)	0.85 (0.75 to 0.95)	0.73 (0.58 to 0.89)

AUC, area under the curve; CI, confidence interval; OR, odd ratio; SARS-CoV-2, severe acute respiratory syndrome coronavirus 2;

^a^Adjusted for age and gender

^b^Adjusted for age, gender, creatinine level, diastolic blood pressure as well as cancer and coronary artery disease

^c^Odds ratio for 1 pmol/L increase of proET-1

The association of proET-1 levels at measured time points with the primary endpoint is shown in Table 4. The highest discrimination performance of proET-1 levels was found at time point 2, i.e., day 5 or 6 of hospitalization (AUC of 0.91). A 10 pmol/L increase of proET-1 at this time point of hospitalization was associated with a 20% higher risk for 30-day mortality in the unadjusted analysis (OR 1.2 (1.0 to 1.4, p = 0.01)). However, this was not significant in the multivariable analysis.

**Table 4:** Logistic regression analyses for different proET-1 cut-offs at different time points. Crude and adjusted association of different proET-1 cut-offs at different time points and 30-day mortality

	Survivors (n = 57)	Non-Survivors (n = 17)	p-value	AUC	Univariable OR^c^ (95% CI), p-value	Multivariabl^a^ OR^c^ (95% CI), p-value	Multivariable^b^ OR^c^ (95% CI), p-value
proET-1 Time point 0 (within 24 h form admission), n = 74							
proET-1 overall, median (IQR)	53.6 (37.0, 69.0)	81.8 (76.0, 118.0)	** < 0.01**	0.74	1.2 (1.1 to 1.4), p < **0.01**	1.1 (0.9 to 1.4), p = 0.12	0.9 (0.6 to 1.2), p = 0.35
proET-1- 74.0-cut-off [pmol/L], n (%)							
≤ 74.0	44 (77%)	4 (24%)	** < 0.01**		Reference	Reference	Reference
> 74.0	13 (23%)	13 (76%)			11.0 (3.1 to 39.6), p < **0.01**	4.8 (1.0 to 22.0), p = 0.05	1.9 (0.3 to 13.0), p = 0.50
proET-1- 94.0-cut-off [pmol/L], n (%)							
≤ 94.0	52 (91%)	12 (71%)	**0.03**		Reference	Reference	Reference
> 94.0	5 (9%)	5 (29%)			4.3 (1.1 to 17.4), p = 0.04	2.6 (0.5 to 14.0), p = 0.27	1.0 (0.1 to 14.0), p = 0.99
proET-1- 154.0-cut-off [pmol/L], n (%)							
≤ 154.0	55 (96%)	15 (88%)	0.19		Reference	Reference	NA
> 154.0	2 (4%)	2 (12%)			3.7 (0.5 to 28.2), p = 0.2	3.3 (0.3 to 38.2), p = 0.34	NA
proET-1- median-cut-off [pmol/L], n (%)							
≤ 106	52 (91%)	12 (71%)	**0.03**		Reference	Reference	Reference
> 106	5 (9%)	5 (29%)			4.3 (1.1 to 17.4), p = **0.04**	2.6 (0.5 to 14.0), p = 0.27	1.0 (0.07 to 14.0), p = 0.99
proET-1 Time point 1 (day 3/day 4 of hospitalization), n = 55							
proET-1 overall, median (IQR)	56.9 (44.5, 77.3)	124.3 (95.4, 156.0)	** < 0.01**	0.81	1.2 (1.0 to 1.04), p < **0.01**	1.2 (1.0 to 1.4), p = **0.03**	1.3 (1.0 to 1.8), p = 0.76
proET-1- 74.0-cut-off [pmol/L], n (%)							
≤ 74.0	30 (70%)	2 (17%)	** < 0.01**		Reference	Reference	Reference
> 74.0	13 (30%)	10 (83%)			11.5 (2.2 to 60.2), p < **0.01**	8.7 (1.5 to 52.3), p = **0.02**	72.0 (1.0 to 5257.0), p = **0.05**
proET-1- 94.0-cut-off [pmol/L], n (%)							
≤ 94.0	37 (86%)	3 (25%)	** < 0.01**		Reference	Reference	Reference
> 94.0	6 (14%)	9 (75%)			18.5 (3.9 to 88.5), p < **0.01**	12.4 (2.2 to 68.3), p < **0.01**	78.6 (1.0 to 5688.0), p = **0.05**
proET-1- 154.0-cut-off [pmol/L], n (%)							
≤ 154.0	41 (95%)	9 (75%)	**0.03**		Reference	Reference	Reference
> 154.0	2 (5%)	3 (25%)			6.8 (0.9 to 47.0), p = 0.05	6.2 (0.7 to 54.3), p = 0.10	68.0 (0.2 to 18,750.0), p = 0.14
proET-1- median-cut-off [pmol/L], n (%)							
≤ 63.4	27 (63%)	1 (8%)	** < 0.01**		Reference	Reference	NA
> 63.4	16 (37%)	11 (92%)			18.6 (2.2 to 157.5), p < **0.01**	17.0 (1.8 to 160.7), p = **0.01**	NA
proET-1 Time point 2 (day 5/day 6 of hospitalization), n = 45							
proET-1 overall, median (IQR)	54.0 (39.0, 91.2)	163.2 (107.2, 217.6)	** < 0.01**	0.91	1.2 (1.0 to 1.4), p = **0.01**	1.1 (1.0 to 1.3), p = **0.03**	N/A
proET-1- 74.0-cut-off [pmol/L], n (%)							
≤ 74.0	26 (72%)	1 (11%)	** < 0.01**		Reference	Reference	NA
> 74.0	10 (28%)	8 (89%)			20.8 (2.3 to 188.3), p < **0.01**	19.3 (1.6 to 238.0), p = **0.02**	NA
proET-1- 94.0-cut-off [pmol/L], n (%)							
≤ 94.0	28 (78%)	1 (11%)	** < 0.01**		Reference	Reference	NA
> 94.0	8 (22%)	8 (89%)			28.0 (3.0 to 258.4), p < **0.01**	25.2 (2.01 to 307.2), p = **0.01**	NA
proET-1- 154.0-cut-off [pmol/L], n (%)							
≤ 154.0	33 (92%)	4 (44%)	** < 0.01**		Reference	Reference	Reference
> 154.0	3 (8%)	5 (56%)			13.8 (2.3 to 80.6), p < **0.01**	9.8 (1.4 to 70.3), p = **0.02**	15.6 (0.5 to 499.2), p = 0.12
proET-1- median-cut-off [pmol/L], n (%)							
≤ 59.5	23 (64%)	0 (0%)	** < 0.01**		NA	NA	NA
> 59.5	13 (36%)	9 (100%)			NA	NA	NA
proET-1 Time point 3 (day 7/day 8 of hospitalization), n = 31							
proET-1 overall, median (IQR)	56.5 (38.6, 101.7)	108.6 (97.8, 169.4)	**0.04**	0.8	1.1 (0.9 to 1.3), p = 0.06	1.1 (1.0 to 1.3), p = 0.12	1.2 (0.9 to 1.5), p = 0.16
proET-1- 74.0-cut-off [pmol/L], n (%)							
≤ 74.0	15 (58%)	0 (0%)	**0.02**		NA	NA	NA
> 74.0	11 (42%)	5 (100%)			NA	NA	NA
proET-1- 94.0-cut-off [pmol/L], n (%)							
≤ 94.0	17 (65%)	1 (20%)	0.06		Reference	Reference	Reference
> 94.0	9 (35%)	4 (80%)			7.6 (0.7 to 78.1), p = 0.09	4.6 (0.4 to 54.7), p = 0.23	11.3 (0.2 to 593.4), p = 0.23
proET-1- 154.0-cut-off [pmol/L], n (%)							
≤ 154.0	23 (88%)	3 (60%)	0.11		Reference	Reference	Reference
> 154.0	3 (12%)	2 (40%)			5.1 (0.6 to 44.1), p = 0.14	2.6 (0.2 to 26.7), p = 0.43	3.6 (0.1 to 86.5), p = 0.43
proET-1- median-cut-off [pmol/L], n (%)							
≤ 81.65	16 (62%)	0 (0%)	**0.01**		NA	NA	NA
> 81.65	10 (38%)	5 (100%)			NA	NA	NA

Bold values indicate statistical significant

AUC, area under the curve; CI, confidence interval; NA, not applicable; OR, odd ratio; proET-1, proEndothelin-1

^a^Adjusted for age and gender

^b^Adjusted for age, gender, creatinine level, diastolic blood pressure as well as cancer and coronary artery disease

^c^Odds ratio for 10 pmol/L increase of proET-1

### Prognostic accuracy of proET-1 in COVID-19 at specific cut-off levels

Prognostic accuracy of proET-1 to predict 30-day mortality was analyzed for specific proET-1 cut-offs (Table 5). Based on the Youden-Index, we found an optimal cut-off at 74 pmol/l, where the sensitivity to correctly predict 30-day all-cause mortality was 76.5% (95% CI 50.1 to 93.2) with a specificity of 77.2% (95% CI 64.2 to 87.3%). Furthermore, the cut-off at 74 pmol/l showed a high negative predictive value of 91.7% (95% CI 80.0 to 97.7). The 94 pmol/L cut-off showed a high specificity of 91.2% (corresponding sensitivity of 29.4%).

**Table 5:** Prognostic accuracy of different proET-1 cut-offs at baseline for patients with COVID-19

*ProET-1 Cut-off value [pmol/l]*	Sensitivity (95% CI)	Specificity (95% CI)	Positive predictive value (95% CI)	Negative predictive value (95% CI)
74.0 pmol/l	76.5 (50.1 to 93.2)	77.2 (64.2 to 87.3)	50.0 29.9 to70.1)	91.7 (80.0 to 97.7)
94.0 pmol/l	29.4 (10.3 to 56.0)	91.2 (80.7 to 97.1)	50.0 (18.7 to 81.3)	81.3 (69.5 to 89.9)
154.0 pmol/l	11.8 (1.5 to 36.4)	96.5 (87.9 to 99.6)	50.0 (6.8 to 93.2)	78.6 (67.1 to 87.5)
ProET-1 median pmol/l]				
107.0 pmol/l	29.4 (10.3 to 56.0)	91.2 (80.7 to 97.1)	50.0 (18.7 to 81.3)	81.3 69.5 to 89.9)

CI, confidence interval; proET-1, proEndothelin-1

## Discussion

This prospective study evaluated endothelial activation through measurement of initial proET-1 in patients with confirmed COVID-19, as well as patients with other types of respiratory infections. Although we expected an increase in proET-1 in COVID-19 patients compared to other types of respiratory infections, our results showed quite the opposite. In fact, our results indicate a less pronounced activation of proET-1 in COVID-19 compared to other respiratory tract infections with non-significant associations with mortality in multivariable analyses. Although proET-1 provided some prognostic information regarding mortality, discrimination analysis based on C-statistic showed only a moderate prognostic accuracy. These results suggest that in COVID-19 patients proET-1 is not a powerful marker for calculating morbidity and mortality in contrast to its validity in other respiratory infections.

Several pro-inflammatory cytokines and prognostic markers from different pathways have been investigated in patients with COVID-19 [30, 31]. Yet, to our knowledge, there is a lack of data looking at endothelial biomarkers including proET-1 in patients affected by COVID-19.

There is a strong rationale for looking at proET-1 in COVID-19 patients based on results of previous research in other types of infections. More specifically, the prognostic relevance of proET-1 was analyzed in several prior studies focusing on critical-ill patients with and without sepsis [14], patients with septic shock [32], patients with myocardial injury and myocardial dysfunction due to septic shock [33], as well as patients with community acquired pneumonia [29]. For critically ill patients, results showed elevated proET-1 as an independent risk factor for ICU admission and overall mortality regardless of sepsis diagnosis [14]. Another study found an about eightfold increase of proET-1 in the plasma of septic patients with increasing severity of infection [32] and showed that proET-1 correlates with disease severity. Further, it can be used as independent predictor for mortality in patients with community-acquired pneumonia. However, our results suggest that proET-1 may not be as important in COVID-19 compared to other infections. In fact, our results showed that patients with COVID-19 had lower initial proET-1 levels, when compared to patients with pneumonia and exacerbated bronchitis. Clinically, COVID-19 initially has less effect on the (cardio) vascular system but is more affecting the lungs. COVID-19 patients rarely need vasopressor support despite high severity of illness, while in sepsis hypotension and shock is a hallmark of the disease. This may be explained by the fact that COVID-19 is a systemic disease that affects many organs, especially different organs and tissues, where the ACE2 receptors are expressed allowing the virus to enter the cells. Some authors report that the density of ACE2 in each tissue may correlate with the severity of COVID-19 in that tissue [7, 34–38]. However, other than in sepsis, patients with COVID-19 remain hemodynamically stable and therefore, proET-1 and ET-1 remain low. Another reason might be, that SARS-CoV-2 enters endothelial cells via ACE2 receptors and leads to cell damage that stops them from releasing proET-1, as already described in a similar way for SARS-CoV when entering pancreatic islet cells and suppressing or destroying them [39].

In our study population, COVID-19 patients were mainly transferred to ICU due to the need for non-invasive and/or invasive ventilation support. In contrast, reason for ICU admission in patients with other respiratory infection is often due to shock and need for vasopressor support. Other than in septic patients, where the need of pressure support and rising levels of the vasoconstrictor ET-1 are crucial, the reason for severe disease and death in patients with COVID-19 may rather be related to the infection-mediated endothelial injury and endothelialitis. This in turn may trigger excessive thrombin production, inhibit fibrinolysis, and activate complement pathways, which consequently initiate thrombo-inflammation and can lead to micro-thromboembolism and microvascular dysfunctions [40].

Compared to prior studies [14], the optimal cut-off for the prediction of 30-day mortality found in our study was lower with a value of 74 pmol/L. This cut-off showed the best sensitivity and specificity to predict all-cause 30-day mortality in patients with COVID-19. The use of higher cut-offs like in prior studies [29], showed only a very low sensitivity.

## Limitations

This study has several limitations. The results of this analysis have to be interpreted in the context of the study design: First, due to the single center design of this study the number of analyzed COVID-19 cases was small and external validation is needed. Second, clinical data were limited and not all evaluated laboratory parameters and characteristics were available for all patients, resulting in few missing data. Third, due to incomplete data, left atrial size, which represent a known confounder of proET-1 levels, was not considered in the adjusted regression models.

## Conclusion

In conclusion, as compared to other types of pulmonary infection, COVID-19 causes increases of proET-1 concentrations to a lesser extent, which might be explained by either only a mild activation of the endothelium or by the reduced possibility to produce the hormone from damaged endothelium. Thus, we could not find any evidence that the high mortality associated with COVID-19 can be estimated by the endothelial function marker proET-1. Based on our results, the use of proET-1 for prognostic risk stratification in patients with COVID-19 is not recommended.

## Data Availability

The datasets used and analyzed during the current study are available from the corresponding author on reasonable request.

## Abbreviations

ACE2: Angiotensin-converting enzyme 2
BMI: Body mass index
bpm: beats per minute
C: Celsius
CI: Confidence intervals
COVID-19: Coronavirus disease 2019
CRP: C-reactive protein
ET-1: Endothelin-1
ICD-10: International statistical classification of diseases and related health problems codes
ICU: Intensive care unit
IQR: Interquartile ranges
LOD: Limit of detection
LOS: Length of hospital stay
LRTI: Lower respiratory tract infection
NS: Non-survivors
proET-1: ProEndothelin-1
OR: Odds ratios
RAAS: Renin–angiotensin–aldosterone system
ROC-AUC: Area under the operating receiver curve
RT-PCR: Real-time reverse transcription polymerase chain reaction
S: Survivors
SARS-CoV-2: Severe acute respiratory syndrome coronavirus 2
SD: Standard deviation
TNF-alpha: Tumor necrosis factor-alpha
WHO: World health organization
